# Retinal microvascular associations with cardiometabolic risk factors differ by diabetes status: results from the UK Biobank

**DOI:** 10.1007/s00125-022-05745-y

**Published:** 2022-07-19

**Authors:** Robyn J. Tapp, Christopher G. Owen, Sarah A. Barman, David P. Strachan, Roshan A. Welikala, Paul J. Foster, Peter H. Whincup, Alicja R. Rudnicka, on behalf of the UK Biobank Eyes and Vision Consortium

**Affiliations:** 1 Population Health Research Institute, St George’s, University of London, London, UK; 2 Research Centre for Intelligent Healthcare, Coventry University, Coventry, UK; 3 Faculty of Science, Engineering and Computing, Kingston University, Kingston upon Thames, Surrey, UK; 4 Integrative Epidemiology Research Group, UCL Institute of Ophthalmology, London, UK; 5 NIHR Biomedical Research Centre at Moorfields Eye Hospital, London, UK

**Keywords:** Cardiometabolic risk, Diabetes, Diameters, Epidemiology, Retinal imaging, Retinal microvasculature, Tortuosity

## Abstract

**Aims/hypothesis:**

The aim of the study was to examine the association of retinal vessel morphometry with BP, body composition and biochemistry, and to determine whether these associations differ by diabetes status.

**Methods:**

The UK Biobank ocular assessment included 68,550 participants aged 40-70 years who underwent non-mydriatic retinal photography, BP and body composition measurements, and haematological analysis. A fully automated image analysis program provided measurements of retinal vessel diameter and tortuosity. The associations between retinal vessel morphology and cardiometabolic risk factors by diabetes status were examined using multilevel linear regression, to provide absolute differences in vessel diameter and percentage differences in tortuosity (allowing for within-person clustering).

**Results:**

A total of 50,233 participants (a reduction from 68,550) were included in these analyses. Overall, those with diabetes had significantly more tortuous venules and wider arteriolar diameters compared with those without. Associations between venular tortuosity and cardiometabolic risk factors differed according to diabetes status (*p* interaction <0.01) for total fat mass index, HbA_1c_, C-reactive protein, white cell count and granulocyte count. For example, a unit rise in white cell count was associated with a 0.18% increase (95% CI 0.05, 0.32%) in venular tortuosity for those without diabetes and a 1.48% increase (95% CI 0.90, 2.07%) among those with diabetes. For arteriolar diameter, significant interactions were evident for systolic BP, diastolic BP, mean arterial pressure (MAP) and LDL-cholesterol. For example, a 10 mmHg rise in systolic BP was associated with a −0.92 μm difference (95% CI −0.96 to −0.88 μm) in arteriolar diameter for those without diabetes, and a −0.58 μm difference (95% CI −0.76 to −0.41 μm) among those with diabetes. No interactions were observed for arteriolar tortuosity or venular diameters.

**Conclusions/interpretation:**

We provide clear evidence of the modifying effect of diabetes on cardiometabolic risk factor associations with retinal microvascular architecture. These observations suggest the occurrence of preclinical disease processes, and may be a sign of impaired autoregulation due to hyperglycaemia, which has been suggested to play a pivotal role in the development of diabetes-related microvascular complications.

**Data Availability:**

The data supporting the results reported here are available through the UK Biobank (https://www.ukbiobank.ac.uk/enable-your-research/apply-for-access).

**Graphical abstract:**

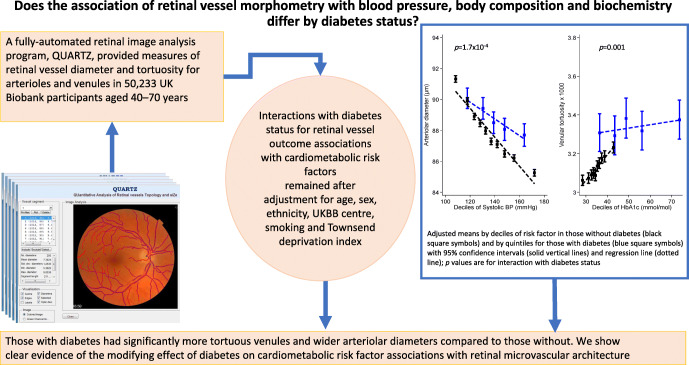

**Supplementary Information:**

The online version contains peer-reviewed but unedited supplementary material available at 10.1007/s00125-022-05745-y.

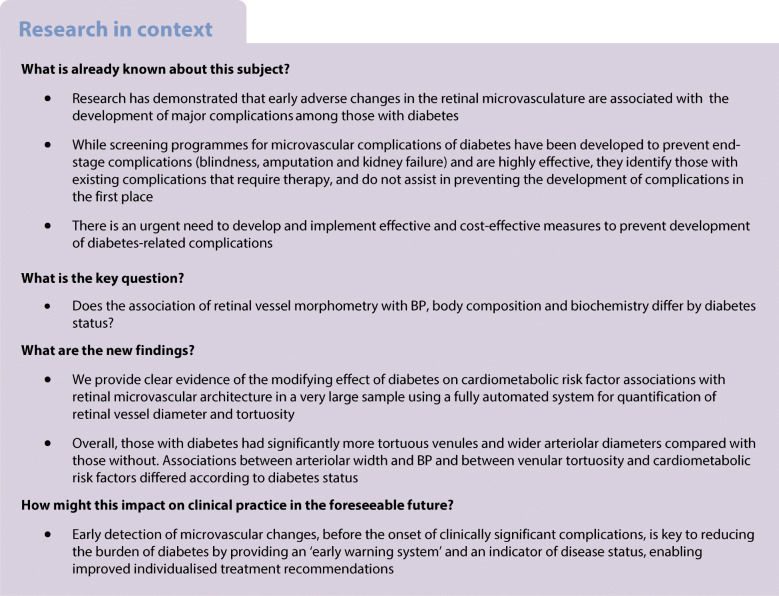



## Introduction

Type 2 diabetes globally affects an estimated 425 million people [1], and imposes a large economic burden on individuals, their families and national health systems. The global cost of diabetes has been estimated at US$1.3 trillion, with the largest contributor to costs being treatment of diabetes-related complications [1]. There is an urgent need to develop and implement effective and cost-effective measures to prevent diabetes and its associated complications.

Early detection of microvascular changes, well before the onset of clinically significant diabetes-related complications, is key to reducing the burden of diabetes by providing an ‘early warning system’ and an indicator of disease status, enabling improved individualised treatment recommendations. The significance of this novel approach becomes clear when considering key limitations to our current management and screening practices for diabetes and its complications. Patient-appropriate HbA_1c_ targets are a cornerstone of diabetes management, due to their strong association with risk of developing microvascular complications [2]; however, HbA_1c_ does not always reflect or predict the burden of disease, and fails to account for hypoglycaemia and glucose variability [3, 4]. While screening programmes for microvascular complications have been developed to prevent end-stage complications (blindness, amputation and kidney failure) and are highly effective [5–7], they identify those with existing complications, requiring therapy, and do not assist in preventing development of complications that, at the time of diagnosis, are unlikely to be reversible.

Recent research has successfully demonstrated that early adverse changes in the retinal microvasculature are associated with a myriad of cardiometabolic risk factors in the general population [8–13], and with major complications among those with diabetes [14]. These associations are important, as the capacity for the microvasculature to revert towards ‘normal’ following intensive management strongly suggests plasticity early in the disease course [15–17]. However, adverse changes in the retinal microvasculature with an increased dose response to cardiometabolic risk factors (factors that are also key in the development of diabetes complications) are complex, and while consistent associations have been observed in the general population [11, 18–20], the results have been mixed among those with diabetes [14]. It appears that, rather than proceeding along a continuum, once diabetes is established, the impact of cardiometabolic risk factors on the retinal microvasculature may become blunted or less pronounced, i.e. the expected associations are attenuated and the vascular physiology becomes incapable of responding to the normal physiological stimuli. However, the results have been variable, and the population-based studies that have been performed are too small to confirm them [14, 21, 22]. These changes in associations have been linked with incident diabetes [14] as well as both microvascular complications of diabetes (diabetic retinopathy, nephropathy and neuropathy) [14, 23, 24] and macrovascular complications of diabetes (stroke, myocardial infarction and hypertension) [25, 26].

With the development of fully automated retinal processing software, we now have the capacity to fully assess these associations at scale, and perform definitive assessments of the risk factor associations that may prove useful in developing a clinical risk prediction tool for diabetes complications. We determined the association of cardiometabolic risk factors with the retinal microvasculature among participants with and without diabetes in the UK Biobank.

## Methods

The UK Biobank was set up as a national resource to allow researchers to investigate and develop techniques aimed at improving the prevention, diagnosis and treatment of a wide range of non-communicable diseases. The study recruited more than 500,000 people aged 40-70 years between 2006 and 2010 from across the UK. A subset of 68,550 participants had retinal images captured, providing 135,867 macula-centred retinal images (from both eyes). In the present study, those with incomplete data for age, sex, BMI and systolic BP were excluded in line with our previous reports [12, 13], and an additional 4477 were excluded for having incomplete biochemistry data (triacylglycerols, total cholesterol and LDL-cholesterol). Colour images were captured using a non-mydriatic fundus camera (Topcon 3D OCT-1000 Mk2, Japan) with a 45° field of view, and saved in PNG format with a resolution of 2048 × 1536 pixels.

### Physical examination

Participants underwent a physical examination, with age, sex, smoking status, medication use and other sociodemographic characteristics being collected using a computerised questionnaire. At a postcode level, neighbourhood deprivation was expressed in terms of the Townsend deprivation index. Height was measured to the nearest cm (Seca 202 stadiometer, Seca, Hamburg, and a body composition analyser was used to measure weight to the nearest 0.1 kg and to obtain other measurements of body size (Tanita Body Composition Analyser BC-418MA, Tanita, Japan). BMI was calculated as weight (kg) divided by height (m^2^). Body size measurements included total fat mass index (TFMI) and total fat-free mass index. Two BP measurements were taken using an HEM-7015IT electronic BP monitor (Omron Healthcare, the Netherlands), at least 1 min apart, in a seated position [27]. Mean arterial pressure (MAP) was calculated as [(2 × diastolic BP) + systolic BP]/3. History of myocardial infarction, stroke and diabetes (type 1 or type 2) were determined by self-reporting at the baseline examination. The HbA_1c_ assay was performed using five Variant II Turbo analysers( Bio-Rad Laboratories, USA). Additional measurements included triacylglycerol, C-reactive protein (CRP), white cell count (WCC) and granulocyte count. Analysis of serum biomarkers used ten immunoassay analysers (six Liaison XL DiaSorin Italy and four DXI 800 analysers, Beckman Coulter Diagnostics, Switzerland) and four clinical chemistry analysers (two AU5800 Beckman Coulter Diagnostics and two Advia 1800 analysers Siemens, Germany; https://www.ukbiobank.ac.uk/enable-your-research/about-our-data/biomarker-data). Arterial stiffness was measured non-invasively using the pulse waveform obtained at the finger with an infra-red sensor (Pulse Trace PCA2, CareFusion, USA) [28]. The participant's height (m) was divided by the time between the peaks of the pulse waveform to obtain the arterial stiffness index.

### Retinal imaging and processing

Retinal images were processed using a fully automated computerised system: quantitative analysis of retinal vessel topology and size (QUARTZ) [29]. In brief, this fully automated system [29, 30] obtained thousands of measurements of width and tortuosity from the whole retinal image (dependent on image quality) [31]. These measurements were summarised using mean width and tortuosity, weighted by vessel segment length, for arterioles and venules separately for each image. The system performance has been outlined in detail and validated previously, and allows automated processing of images from large population-based studies [32]. The following image processing modules were all validated on a subset of 4692 retinal images from a random sample of 2346 UK Biobank participants: vessel segmentation, image quality score, optic disc detection, vessel width measurement, tortuosity measurement and arteriolar/venular recognition [29, 30, 33]. The performance of the arteriole/venule recognition module showed detection rates of up to 96% for arterioles and 98% for venules when the automated probability of artery or vein was set to a cut-off of 0.8. An automated assessment of image quality was also performed based on the segmented vasculature [30]. The algorithm achieved a sensitivity of 95.3% and a specificity of 91.1% for detection of inadequate images [33]. A model eye was used to quantify the magnification characteristics of the Topcon 3D OCT-1000 Mk2 fundus camera, allowing pixel dimensions of vessel diameter to be converted into real size [34].

### Ethical approval

The UK Biobank study was approved by the Northwest Region NHS research ethics committee, and the reported investigations have been performed in accordance with the principles of the Declaration of Helsinki as revised in 2008.

### Statistical analysis

The data analysis was performed using Stata 15.0 SE (Stata Corp, College Station, TX, USA). Histograms of retinal vessel widths showed normal distributions, while measurements of tortuosity were positively skewed and log-transformed. Data missing for categorical variables were included as an additional category for each variable to minimise data loss. Multilevel linear regression models, allowing for repeated measurements of vessel indices within the same person, adjusted for age, sex, ethnicity and UK Biobank centre (model 1), were used to examine absolute differences in retinal vessel outcomes by diabetes status. Model 2 extended model 1 with further adjustment for BMI, smoking, the Townsend deprivation index, systolic BP, total cholesterol and triacylglycerols. Model 3 further adjusted model 2 for CRP, WCC and granulocyte count. Model 4 included the same factors as model 3, but excluded participants with a history of heart attack, stroke or those on treatment for hypertension. Multilevel linear regression models adjusting for age, sex, ethnicity and UK Biobank centre, smoking status and Townsend deprivation index were also used to assess associations between retinal vessel outcomes and cardiometabolic risk factors while allowing for an interaction between self-reported diabetes status and each cardiometabolic risk factor in turn. The *p* value for interaction was set to <0.01 for statistical significance given the large number of tests and the large sample size.

## Results

The characteristics of the population overall and by diabetes status are shown in Table 1. A total of 50,233 participants were included in these analyses. The mean age overall was 56.1 years (SD 8.2), and 55.3% were female. The median duration of diabetes was 5 years (IQR 2−10 years). Those with diabetes (compared with those without diabetes) were older (59 years vs 56 years), and had consistently higher systolic BP, body composition measurements, inflammatory markers and morbidity (% stroke and heart attack).

**Table 1:** Characteristics of the UK Biobank population by self-reported diabetes status

Variable	All	Without diabetes	With diabetes
Number of participants^a^	50,233	47,505	2402
Age (years)	56.1 (8.2)	56.0 (8.2)	59.0 (7.5)
Sex (% female)	55.3	56.1	59.0
Ethnicity (%)			
White	91.0	91.8	81.4
Black	2.8	2.6	5.7
Asian	2.8	2.5	8.0
Other	2.8	2.7	4.3
Unknown/did not answer	0.7	0.4	0.7
Smoking (%)			
Never smoker	55.7	56.2	48.8
Occasional	2.9	2.9	2.8
Ex-smoker	34.1	33.9	39.5
Current smoker	6.7	6.7	8.1
Prefer not to say/missing	0.6	0.3	0.7
Townsend deprivation index (%)			
<−3.4	24.9	25.2	20.9
−3.4 to −1.6	25.3	25.6	21.1
−1.7 to 0.8	25.1	25.2	24.9
>0.8	24.5	23.9	32.9
Missing	0.1	0.1	0.2
Medication for hypertension (%)	18.8	16.7	59.4
Systolic BP (mmHg)	136.5 (18.3)	136.3 (18.3)	139.6 (16.7)
Diastolic BP (mmHg)	81.7 (10.0)	81.7 (10.0)	81.2 (9.7)
MAP (mmHg)	99.9 (11.8)	99.9 (11.8)	100.6 (10.6)
BMI (kg/m^2^)	27.3 (4.7)	27.1 (4.6)	30.9 (5.8)
TFMI (kg/m^2^)	8.8 (3.6)	8.7 (3.5)	10.7 (4.3)
Total fat-free mass index (kg/m^2^)	18.5 (2.6)	18.4 (2.5)	20.2 (2.8)
HbA_1c_ (mmol/mol)	35.9 (6.5)	35.1 (4.4)	51.8 (14.3)
HbA_1c_ (%)	5.4 (2.7)	5.4 (2.6)	6.9 (3.5)
Total cholesterol (mmol/l)	5.7 (1.1)	5.8 (1.1)	4.5 (1.1)
HDL-cholesterol (mmol/l)	1.5 (0.4)	1.5 (0.4)	1.2 (0.4)
LDL-cholesterol (mmol/l)	3.5 (0.9)	3.6 (0.8)	2.7 (0.8)
Triacylglycerols (mmol/l)	1.7 (1.0)	1.6 (0.9)	2.0 (1.1)
CRP (μmol/l)^b^	1.31 (2.9)	1.3 (2.9)	1.7 (2.9)
WCC (10^9^ cells/l)	7.0 (2.1)	7.0 (2.1)	7.8 (2.1)
Granulocytes (10^9^ cells/l)	1.5 (0.5)	1.5 (0.5)	1.7 (0.5)
Image quality	0.9 (0.1)	0.9 (0.1)	0.9 (0.1)
Arteriolar width (μm)	88.0 (7.8)	87.9 (7.7)	88.7 (8.1)
Venular width (μm)	103.9 (13.1)	103.9 (13.1)	104.4 (14.0)
Arteriolar tortuosity (×10^3^)	4.4 (1.6)	4.4 (1.6)	4.4 (1.7)
Venular tortuosity (×10^3^)	3.2 (1.4)	3.1 (1.4)	3.3 (1.4)
Heart attack (%)	1.8	1.5	6.9
Stroke (%)	1.3	1.2	3.5

Values are means (SD) or %

Data missing for categorical variables have been included as an additional category for each variable to minimise data loss. For continuous variables, the number of participants with missing data were: 4111 for HbA_1c_, 2421 for glucose, 2373 for HDL, 123 for CRP, 1113 for WCC, 1113 for granulocyte count

^a^Missing values for diabetes status *n*=326

^b^CRP values represent the exponentiated geometric mean and SD

### Absolute differences in retinal diameters and tortuosity by diabetes status

There was no difference in arteriolar tortuosity between those with and without diabetes (Table 2). However, the venules of those with diabetes were 2.72% more tortuous (95% CI 1.42, 4.05%) after adjustment for age, sex, ethnicity, UK Biobank centre, BMI, smoking, the Townsend deprivation index, systolic BP, total cholesterol and triacylglycerols (model 2). Further adjustment for CRP, WCC and granulocyte count (model 3) had little impact, but excluding those with a history of heart attack, stroke and medication for hypertension (model 4) reduced the difference between those with and without diabetes for venular tortuosity. Those with diabetes had 0.63 μm wider arterioles (95% CI 0.32, 0.95 μm), compared with those without diabetes after the adjustments in model 2). The further adjustments in models 3 and 4 had little impact on these differences. There was no clear evidence for any differences in venular diameter between those with and without diabetes.

**Table 2:** Percentage differences in tortuosity and absolute differences in diameter among those with diabetes compared with those without diabetes

Model	Percentage difference in tortuosity				Absolute difference in diameters			
	Arteriolar tortuosity	*p* value	Venular tortuosity	*p* value	Arteriolar diameter	*p* value	Venular diameter	*p* value
Model 1: adjusted for age, sex, ethnicity and UK Biobank centre	1.39 (-0.66, 3.48)	0.186	4.81 (3.53, 6.11)	7.80 × 10^-14^	0.72 (0.41, 1.03)	5.0 × 10^-06^	0.58 (0.05, 1.11)	0.033
Model 2: adjusted as for model 1 + BMI, smoking, Townsend deprivation index, systolic BP, total cholesterol and triacylglycerols	1.25 (-0.88, 3.43)	0.252	2.72 (1.42, 4.05)	3.96 × 10^-05^	0.63 (0.32, 0.95)	7.8 × 10^-05^	0.01 (-0.54, 0.56)	0.974
Model 3: adjusted as for model 2 + CRP, WCC and granulocyte count	1.59 (-0.58, 3.81)	0.153	2.51 (1.18, 3.85)	1.92 × 10^-04^	0.62 (0.30, 0.94)	1.3 × 10^-04^	0.03 (-0.53, 0.59)	0.918
Model 4: model 3, excluding those with history of heart attack, stroke or on medication for hypertension	-0.70 (-3.85, 2.56)	0.670	1.48 (-0.46, 3.47)	0.136	0.98 (0.50, 1.46)	1.1 × 10^-04^	0.06 (-0.78, 0.88)	0.879

Values are percentage differences in retinal vessel tortuosity and absolute differences in diameter from a multilevel model allowing for repeated images from the same person (random effect for person), with 95% CI in parentheses

### Association of retinal vessel tortuosity with cardiometabolic risk: interactions with diabetes status

**Fig. 1:**
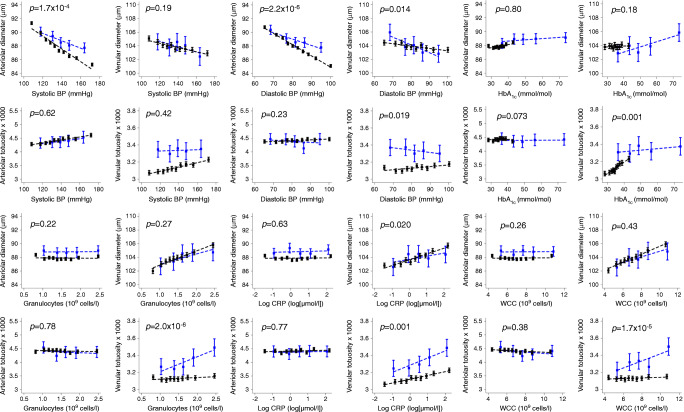
Adjusted mean retinal vessel width and tortuosity by deciles of cardiometabolic risk markers in those without diabetes and by quintiles in those with diabetes, for factors with at least one interaction value with a *p* value <0.01. The adjusted means (solid square symbols), 95% CI (solid vertical error bars) and regression line (dotted lines) were obtained from a multilevel model allowing for age and sex as fixed effects and repeated retinal vessel measurements within each person. Blue lines and symbols represent those with diabetes; black lines and symbols represent those without diabetes. The *p* values are for interactions in linear associations of cardiometabolic risk markers with diabetes status

No interactions were observed between diabetes status and cardiometabolic risk factor associations with arteriolar tortuosity (*p* interaction >0.01). For venular tortuosity, associations with cardiometabolic risk factor differed by diabetes status: *p* values for interactions were statistically significant for TFMI, HbA_1c_, CRP, WCC and granulocyte count with adjustment (Table 3). For example, a unit rise in HbA_1c_ was associated with a 1.23% increase in venular tortuosity in those without diabetes (95% CI 0.90, 1.56%) and a 0.32% increase in venular tortuosity among those with diabetes (95% CI −0.12, 0.76%), and a unit rise in WCC (×10^9^cells/l) was associated with a 0.18% increase in venular tortuosity among those without diabetes (95% CI 0.05, 0.32%) and a 1.48% increase in venular tortuosity among those with diabetes (95% CI 0.90, 2.07%). The associations of venular tortuosity with TFMI, CRP, WCC and granulocyte count were in the same direction among those with and without diabetes, but a unit rise in these risk markers was associated with a larger increase in venular tortuosity in those with diabetes compared with those without diabetes (Fig. 1).

**Table 3:** Cross-sectional associations showing percentage difference in retinal tortuosity per specified rise in CVD risk factors, by diabetes status

	Percentage difference in arteriolar tortuosity			Percentage difference in venular tortuosity		
Risk marker	Without diabetes	With diabetes	*p* interaction	Without diabetes	With diabetes	*p* interaction
Age, per decade	2.39 (1.83, 2.94)	1.12 (-1.51, 3.76)	0.356	2.43 (2.10, 2.76)	2.79 (1.20, 4.37)	0.667
Systolic BP, per 10 mmHg	1.24 (0.97, 1.50)	0.92 (-0.26, 2.12)	0.615	0.63 (0.47, 0.79)	0.33 (-0.39, 1.04)	0.415
Diastolic BP, per 10 mmHg	0.90 (0.44, 1.35)	-0.38 (-2.39, 1.67)	0.232	0.35 (0.08, 0.62)	-1.16 (-2.36, 0.07)	0.019
MAP, per 10 mmHg	1.37 (0.97, 1.76)	0.48 (-1.36, 2.36)	0.363	0.64 (0.41, 0.88)	-0.41 (-1.51, 0.71)	0.072
BMI, per 5 kg/m^2^	0.05 (-0.44, 0.55)	1.22 (-0.48, 2.96)	0.197	2.21 (1.91, 2.51)	3.06 (2.01, 4.11)	0.127
TFMI, kg/m^2^	-0.01 (-0.69, 0.67)	1.90 (0.13, 3.71)	0.033	1.78 (1.37, 2.20)	3.20 (2.12, 4.30)	0.010
Total fat-free mass index, kg/m^2^	0.05 (-0.85, 0.96)	0.52 (-1.46, 2.53)	0.635	0.84 (0.29, 1.38)	0.65 (-0.54, 1.86)	0.756
HbA_1c_, per 5 mmol/mol	0.90 (0.35, 1.45)	0.07 (-0.65, 0.79)	0.073	1.23 (0.90, 1.56)	0.32 (-0.12, 0.76)	0.001
Total cholesterol, mmol/l	0.18 (-0.24, 0.59)	1.31 (-0.58, 3.23)	0.251	-0.35 (-0.60, -0.11)	0.16 (-0.97, 1.30)	0.388
HDL-cholesterol, mmol/l	0.62 (-0.67, 1.93)	1.17 (-4.38, 7.03)	0.854	-1.78 (-2.54, -1.02)	-0.05 (-3.39, 3.40)	0.322
LDL-cholesterol, mmol/l	0.00 (-0.53, 0.53)	1.46 (-1.08, 4.07)	0.272	-0.28 (-0.59, 0.04)	0.27 (-1.26, 1.81)	0.498
Triacylglycerol, mmol/l	0.80 (0.31, 1.29)	0.87 (-0.85, 2.62)	0.938	0.12 (-0.17, 0.41)	0.11 (-0.93, 1.16)	0.984
CRP, μmol/l	0.39 (-0.04, 0.82)	0.11 (-1.72, 1.97)	0.773	1.21 (0.95, 1.47)	3.17 (2.04, 4.32)	0.001
WCC, 10^9^ cells/l	-0.39 (-0.61, -0.16)	-0.82 (-1.76, 0.12)	0.377	0.18 (0.05, 0.32)	1.48 (0.90, 2.07)	1.66 × 10^-05^
Granulocytes, 10^9^ cells/l	-1.75 (-2.70, -0.79)	-2.29 (-5.97, 1.52)	0.782	1.12 (0.54, 1.72)	7.12 (4.67, 9.62)	1.95 × 10^-06^

Values are regression coefficients from a multilevel model allowing for repeated images from the same person (random effect for person) and adjusting each factor for age, sex, ethnicity, UK Biobank centre, smoking and the Townsend deprivation index. This is a combined model including the interaction term. Values in parentheses are 95% CI

### Associations of retinal vessel diameter with cardiometabolic risk markers: interactions with diabetes status

Associations of arteriolar diameter with cardiometabolic risk factors differed according to diabetes status (*p* value for interactions with diabetes status <0.01) for systolic and diastolic BP, MAP and LDL-cholesterol, with adjustment for age, sex, ethnicity, UK Biobank centre, smoking and Townsend deprivation index (Table 4). The association of arteriolar diameter with systolic and diastolic BP, and MAP were in the same direction among those with and without diabetes, but per unit rise in these risk factors was associated with greater difference in arteriolar diameter in those without diabetes compared with those with diabetes (Fig. 1). For example, a 10 mmHg rise in systolic BP was associated with a −0.92 μm difference in arteriolar diameter for those without diabetes (95% CI −0.96, −0.88 μm) and a −0.58 μm difference among those with diabetes (95% CI −0.76, −0.41 μm), and a 10 mmHg rise in diastolic BP was associated with −1.65 μm difference in arteriolar diameter among those without diabetes (95% CI −1.71, −1.58 μm) and a −0.90 μm difference among those with diabetes (95% CI −1.20, −0.60 μm). A similar pattern of association was evident for MAP. For LDL, the associations with arteriolar diameter are also inverse, but a unit increase in LDL was associated with greater decrease in arteriolar diameter in those with diabetes compared with those without diabetes. No interactions were observed between diabetes status and WCC, granulocyte count or body composition measurements for arteriolar diameters (*p* interaction >0.01). No interactions were observed for associations of cardiometabolic risk factors with venular diameter associations by diabetes status (*p* interaction >0.01).

**Table 4:** Cross-sectional associations showing difference in retinal diameters per specified rise in CVD risk factors, by diabetes status

	Absolute difference in arteriolar diameter (μm)			Absolute difference in venular diameter (μm)		
Risk marker	Without diabetes	With diabetes	*p* interaction	Without diabetes	With diabetes	*p* interaction
Age, per decade	-0.57 (-0.65, -0.48)	-0.26 (-0.66, 0.14)	0.140	1.02 (0.87, 1.16)	0.74 (0.06, 1.42)	0.437
Systolic BP, per 10 mmHg	-0.92 (-0.96, -0.88)	-0.58 (-0.76, -0.41)	1.74 × 10^-04^	-0.23 (-0.30, -0.16)	-0.44 (-0.74, -0.13)	0.189
Diastolic BP, per 10 mmHg	-1.65 (-1.71, -1.58)	-0.90 (-1.20, -0.60)	2.17 × 10^-06^	-0.22 (-0.34, -0.11)	-0.90 (-1.42, -0.37)	0.014
MAP, per 10 mmHg	-1.51 (-1.57, -1.45)	-0.94 (-1.21, -0.67)	5.29 × 10^-05^	-0.28 (-0.38, -0.18)	-0.84 (-1.32, -0.36)	0.025
BMI, per 5 kg/m^2^	-0.34 (-0.41, -0.26)	-0.31 (-0.57, -0.06)	0.859	0.63 (0.51, 0.76)	0.17 (-0.27, 0.60)	0.045
TFMI, kg/m^2^	0.01 (-0.09, 0.11)	0.10 (-0.16, 0.37)	0.491	0.77 (0.59, 0.94)	0.49 (0.03, 0.94)	0.223
Total fat-free mass index, kg/m^2^	-0.58 (-0.72, -0.45)	-0.62 (-0.92, -0.32)	0.805	-0.13 (-0.36, 0.11)	-0.57 (-1.08, -0.06)	0.078
HbA_1c_, per 5 mmol/mol	0.06 (-0.02, 0.14)	0.08 (-0.03, 0.19)	0.802	0.22 (0.08, 0.36)	0.38 (0.19, 0.56)	0.181
Total cholesterol, mmol/l	-0.20 (-0.27, -0.14)	-0.52 (-0.80, -0.23)	0.035	0.04 (-0.06, 0.15)	0.01 (-0.47, 0.50)	0.907
HDL-cholesterol, mmol/l	-0.75 (-0.94, -0.55)	-0.93 (-1.78, -0.08)	0.684	-1.68 (-2.02, -1.35)	-0.67 (-2.12, 0.79)	0.178
LDL-cholesterol, mmol/l	-0.14 (-0.22, -0.06)	-0.68 (-1.06, -0.29)	0.007	0.20 (0.06, 0.34)	0.01 (-0.64, 0.67)	0.589
Triacylglycerols, mmol/l	-0.17 (-0.24, -0.10)	0.06 (-0.20, 0.33)	0.089	0.44 (0.32, 0.57)	0.29 (-0.16, 0.73)	0.512
CRP, μmol/l	-0.01 (-0.07, 0.06)	0.06 (-0.22, 0.34)	0.633	0.87 (0.76, 0.98)	0.30 (-0.18, 0.77)	0.020
WCC, 10^9^ cells/l	0.00 (-0.04, 0.03)	0.08 (-0.06, 0.23)	0.255	0.36 (0.31, 0.42)	0.26 (0.02, 0.51)	0.430
Granulocytes, 10^9^ cells/l	0.02 (-0.13, 0.16)	0.38 (-0.20, 0.96)	0.225	1.62 (1.37, 1.87)	1.05 (0.06, 2.04)	0.273

Values are regression coefficients from a multilevel model allowing for repeated images from the same person (random effect for person) and adjusting each factor for age, sex, ethnicity, UK Biobank centre, smoking and the Townsend deprivation index. This is a combined model including the interaction term. Values in parentheses are 95% CI

## Discussion

As far as we are aware, this is the first large-scale study to assess the systematic difference in retinal vessel measurements by diabetes status and the modifying effect of diabetes status on the associations between retinal vessel morphometry and key cardiometabolic risk factors. We confirm that those with diabetes tend to have wider arterioles and more tortuous venules. A key finding from this study was that BP associations with arteriolar diameter were attenuated among those with diabetes but associations of LDL-cholesterol with arteriolar diameter were stronger compared with those without diabetes, while venular tortuosity associations with TFMI, CRP, WCC and granulocyte count were stronger, and associations with HbA_1c_ were weaker, among those with diabetes compared with those without diabetes.

### Retinal vessel tortuosity and diameters by diabetes status

In the present study, those with diabetes had more tortuous venules compared with those without diabetes, and this difference was not explained by adjustment for key cardiometabolic risk factors (Table 2). Previous literature has been limited on this point, and the results have been inconsistent, with some studies showing more tortuous venules among those with diabetes [11, 35], while another study showed no difference in venular tortuosity [36] among those with and without diabetes. In agreement with the current study, the European Prospective Investigation into Cancer (EPIC) study of 5942 participants (including 238 with diabetes) showed that those with diabetes had more tortuous venules compared with those without diabetes [11]. Consistent with this, a small clinic-based study by Sasongko et al, which included 327 participants (224 with diabetes and 103 without diabetes) aged ≥18 years, also showed that those with diabetes had more tortuous arterioles and venules [35]. Our analyses adjusted for a wide range of CVD risk factors (age, sex, BP, BMI, cholesterol, triacylglycerols, anti-hypertensive and lipid-lowering medications). In contrast, a study of people of Asian Malay descent by Cheung et al showed that those with diabetes (*n*=594) were more likely to have straighter (less tortuous) arterioles, with no difference in venular tortuosity compared with those without diabetes (*n*=2141) [36]. They also adjusted for a range of CVD risk factors (sex, mean arterial BP, BMI, total cholesterol and current smoking). The discrepancies in findings may be due to the smaller sample sizes in the previous studies or to characteristics of the population (i.e. differences in diabetes duration, ethnicity, and risk factor profile, including diabetes management and history of CVD events). In the present study once those on hypertension treatment or with a history of a heart attack or stroke were removed, differences in venular tortuosity in those with compared with without diabetes were less pronounced. This is because those with advanced metabolic disease have been removed from the analysis, suggesting these changes were associated with advanced disease and events. The study differences may relate to the disease time course and duration of elevated cardiometabolic risk factors, which may differ between populations. Research has consistently shown that retinal blood flow is reduced among those with diabetes of short duration and then increases over time, possibly to maintain the microvascular integrity [37, 38]. Increased venular tortuosity has been associated with hyperglycaemia-mediated changes [14], with several studies having shown a disturbance in blood flow and loss of endothelial cells and pericytes from the vessel walls [39]. These changes may lead to loss of autoregulatory function and loss of capacity to accommodate fluctuations in hydrostatic pressure, leading to the development of diabetes-related complications [14]. This would fit with the hypothesis that mechanical instability and remodelling may be the mechanisms for the initiation and development of tortuous vessels [40].

The difference in arteriolar diameters by diabetes status observed in the present study supports the previous literature, which has consistently shown those with diabetes have wider arteriolar diameters compared with those without diabetes [36, 41–43]. These changes in arteriolar diameter may occur early in the disease course, as the changes have been consistently shown across studies regardless of the duration of diabetes. It is thought that hyperglycaemia and hypoxia may lead to vasodilation and early vascular changes [14]. While BP was higher, lipids were lower among those with diabetes, but arteriolar diameter remained wider in those with diabetes compared with those without diabetes after adjustment for these factors or exclusion of those on hypertension treatment or with a history of a heart attack or stroke (Table 2). Further adjustment for lipid-lowering therapy did not materially alter the findings. It may be that the full impact of this metabolic cascade cannot adequately be determined by the measurements adjusted for. The effects of LDL-cholesterol and activation of CD36/oxidised LDL receptor warrant further investigation, as it has been shown that CD36 mediates multiple pathways associated with the early pathogenesis and progression of diabetes-related complications in general [44]. In the present study, we did not observe any systematic differences in venular diameter between those with or without a diabetes. This is in contrast to previous research; for example, in the study by Cheung et al [36], those with diabetes had significantly wider venular diameters, as also observed in the study by Nguyen et al [42]. While we have adjusted for ethnicity in the present study, the majority of our participants were of European descent, while the study by Cheung et al comprised an Asian population, and that by Nguyen et al comprised a more diverse population, including white, black, Hispanic and Chinese people. Systematic differences between ethnic groups and medication use may be partially confounding these observations, but it was not possible to explore these interactions here as the number of participants of non-European descent in the present study is limited.

### Retinal vessel morphology associations with cardiometabolic risk markers are modified by diabetes status

In this study, we have confirmed established patterns between retinal vessel diameter and BP, and shown that these associations were attenuated in those with diabetes compared with those without diabetes (Table 4 and electronic supplementary material [ESM] Table 1). Individuals with diabetes may have had pre-existing vascular disease (e.g. vascular sclerosis) or a breakdown in the blood/retinal barriers, resulting in defective autoregulation, which may have limited the change in arteriolar diameter in response to higher levels of BP [45]. Retinal venular tortuosity was overall negatively associated with blood lipids (total cholesterol, LDL-cholesterol and triacylglycerols) but positively associated with HbA_1c_, haematological indices (CRP and granulocyte count), BP (systolic BP, diastolic BP and MAP), and body composition (TFMI) (ESM Table 2), with several associations also being evident for arteriolar tortuosity. However, the associations of venular tortuosity with HbA_1c_, WCC, granulocyte count, CRP and TFMI in the present study were modified by diabetes status, meaning that the slopes were different for those with and without diabetes (Table 3). These differences in associations remained after exclusion of those with a history of heart attack, stroke or on medication for hypertension. Once diabetes is diagnosed, there is a higher likelihood that other comorbidities will be identified and treated. Therefore, the fact that the interactions are still seen among those not treated for hypertension implies that prompter or stricter control of high BP among those with diabetes cannot explain the effect modifications observed. As far as we are aware, this is the first study to assess the modifying effect of diabetes on a range of associations of cardiometabolic risk factors with retinal tortuosity. The fundamental message of the HbA_1c_ venular tortuosity interaction by diabetes status may be twofold. The greater tortuosity among those with diabetes than among those without diabetes and the change in slope are indicative of a loss of autoregulation [14], and the changes in inflammatory marker associations are supportive of this. The strong positive correlation of tortuosity with HbA_1c_ among those without diabetes suggests that the venular changes occur along a continuum, with higher HbA_1c_ within the normal and prediabetes range being associated with increased retinal tortuosity. This linear association is consistent with the known association between higher glucose levels and the development of diabetic retinopathy. In our previous work on the diagnostic criteria for type 2 diabetes, we showed that the association of each glucose measure with retinopathy was linear in several populations, with no evidence of a threshold [46, 47]. Given that changes in the retinal microvascular architecture predict the development of diabetic retinopathy, it is not surprising to find evidence of this linear association among those without diabetes [48]. Once you have diabetes, it appears that the degree of glycaemic control does not influence venular tortuosity much; perhaps the venular tortuosity damage has already been done by the time of clinical diagnosis. In contrast, for venular diameter, the association with HbA_1c_ continues right through the range of glucose values.

### Strengths and limitations

As far as we are aware, the present study is the largest to assess the impact of diabetes on retinal vessel morphometry. Although the present study identified novel associations with retinal tortuosity and confirmed the associations for diameters between those with and without diabetes in terms of absolute differences and CVD risk marker interactions, it is a cross-sectional study. Further research is needed at scale using longitudinal data to determine whether these associations can be replicated, particularly given the mixed findings between cross-sectional and longitudinal studies to date [14]. In particular, research on a large scale to determine the longitudinal impact of trajectories of cardiometabolic risk factors (risk factors that are also key risk factors for diabetes-related complications) on retinal morphology and the development of diabetes-related complications is required if we are to develop risk prediction tools to identify those at high risk of developing diabetes-related microvascular complications within a 5-year window, allowing time for a suitable intervention to be implemented. The QUARTZ software is fully automated, incorporates convolutional neural network (CNN) technology and uses information from all vessels extracted within an image, providing precise measurement. Previous grading systems have only used a section of the retinal image for grading; however, given that our findings are consistent with previous literature, this is unlikely to be a major issue.

### Conclusion

We provide clear evidence of the modifying effect of diabetes on the retinal microvasculature. These observations are indicative of preclinical disease processes, and may be a sign of impaired autoregulation due to hyperglycaemia, changes that have been suggested to play a pivotal role in the development of diabetes-related microvascular complications. Longitudinal investigation on a large scale to determine the usefulness of these non-invasive measures as predictors of diabetes-related complications is warranted.

## Supplementary Information

ESM (PDF 45 kb)

## Data Availability

The data supporting the results reported here are available through the UK Biobank (https://www.ukbiobank.ac.uk/enable-your-research/apply-for-access).

## Abbreviations

CRP: C-reactive protein
MAP: Mean arterial pressure
TFMI: Total fat mass index
WCC: White cell count
