# Supplementary material for: Retinal microvascular associations with cardiometabolic risk factors differ by diabetes status: results from the UK Biobank
Source: Diabetologia. 2022 Jul 19;65(10):1652–63. doi: 10.1007/s00125-022-05745-y (PMC9477904; doi:10.1007/s00125-022-05745-y)
Supplement: Supplementary file 1 — (PDF 45 kb) [file 125_2022_5745_MOESM1_ESM.pdf]

ESM Table 1. Mean difference in arteriolar and venular diameter (μm) associated with CVD risk factors

	Absolute difference in arteriolar diameter (95%CI) in μm				Absolute difference in venular diameter (95%CI) in μm			
	Model 1		Model 2		Model 1		Model 2	
Risk marker		pvalue		pvalue		pvalue		pvalue
Age per decade	-0.57 (-0.65, -0.49)	<1.0E-300	-0.52 (-0.60, -0.44)	9.0E-36	0.97 (0.84, 1.11)	<1.0E-300	1.00 (0.86, 1.14)	<1.0E-300
Sex (female)	-0.21 (-0.34, -0.08)	0.002	-0.22 (-0.35, -0.08)	0.001	-0.73 (-0.96, -0.51)	1.3E-10	-0.55 (-0.77, -0.32)	2.0E-06
Systolic BP per 10mmHg	-0.91 (-0.95, -0.88)	<1.0E-300	-0.91 (-0.95, -0.87)	<1.0E-300	-0.25 (-0.31, -0.18)	1.7E-13	-0.31 (-0.37, -0.24)	3.2E-19
Diastolic BP per 10mmHg	-1.63 (-1.69, -1.56)	<1.0E-300	-1.67 (-1.73, -1.60)	<1.0E-300	-0.28 (-0.39, -0.17)	1.2E-06	-0.45 (-0.57, -0.33)	5.0E-14
Mean arterial pressure per 10 mmHg	-1.50 (-1.56, -1.44)	<1.0E-300	-1.52 (-1.58, -1.47)	<1.0E-300	-0.33 (-0.42, -0.23)	9.1E-11	-0.46 (-0.56, -0.36)	7.6E-19
BMI per 5 kg/m²	-0.31 (-0.38, -0.24)	5.1E-18	-0.30 (-0.37, -0.23)	4.0E-17	0.59 (0.47, 0.71)	5.8E-22	0.60 (0.48, 0.72)	1.1E-22
Total fat mass index kg/m²	0.03 (-0.07, 0.13)	0.595	0.03 (-0.07, 0.13)	0.518	0.74 (0.57, 0.91)	4.6E-17	0.75 (0.58, 0.92)	1.5E-17
Total fat free mass index kg/m²	-0.56 (-0.69, -0.43)	2.1E-16	-0.56 (-0.69, -0.42)	3.1E-16	-0.16 (-0.38, 0.07)	0.183	-0.15 (-0.38, 0.08)	0.198
HbA_{1c} per 5 mmol/mol	0.14 (0.08, 0.19)	1.2E-06	0.17 (0.12, 0.23)	2.3E-09	0.29 (0.19, 0.38)	1.7E-09	0.14 (0.05, 0.24)	0.003
Total cholesterol (mmol/L)	-0.24 (-0.29, -0.18)	3.6E-15	-0.25 (-0.30, -0.19)	2.7E-16	0.02 (-0.08, 0.12)	0.734	0.03 (-0.07, 0.13)	0.555
HDL cholesterol (mmol/L)	-0.85 (-1.04, -0.66)	1.2E-18	-1.22 (-1.42, -1.01)	3.3E-32	-1.76 (-2.09, -1.44)	1.3E-26	-1.22 (-1.56, -0.87)	5.7E-12
LDL cholesterol (mmol/L)	-0.19 (-0.26, -0.11)	1.4E-06	-0.18 (-0.26, -0.10)	3.4E-06	0.16 (0.03, 0.29)	0.016	0.13 (0.00, 0.26)	0.053
Triacylglycerol (mmol/L)	-0.11 (-0.18, -0.04)	0.001	-0.07 (-0.14, 0.00)	0.061	0.49 (0.37, 0.61)	7.7E-16	0.29 (0.16, 0.41)	5.2E-06
CRP (μmol/L)	0.04 (-0.03, 0.10)	0.259	0.15 (0.08, 0.22)	1.5E-05	0.91 (0.80, 1.01)	<1.0E-300	0.76 (0.64, 0.88)	1.1E-35
White cell count (10^9 cells/L)	0.05 (0.02, 0.08)	0.003	0.03 (0.00, 0.07)	0.052	0.42 (0.37, 0.48)	<1.0E-300	0.32 (0.26, 0.38)	5.0E-28
Granulacytes (10^9 cells/L)	0.24 (0.10, 0.37)	0.001	0.16 (0.02, 0.31)	0.027	1.84 (1.61, 2.08)	<1.0E-300	1.42 (1.18, 1.67)	5.5E-30

Absolute difference in retinal vessel diameter are from a multilevel model allowing for repeated images from the same person (random effect for person). Confidence interval (CI). Model 1 adjusting each factor for age, sex, ethnicity and UKBB centre as fixed effects (n=50,233). Model 2 adjusting each factor for model 1 plus body mass index, smoking and the Townsend deprivation index, as fixed effects (n=50,233). *HbA_{1c} model 1 n=46,122 and model 2 n=46,122.

ESM Table 2. Percentage differences in arteriolar and venular tortuosity associated with CVD risk factors

	Percentage difference in arteriolar tortuosity (95%CI)				Percentage difference in venular tortuosity (95%CI)			
	Model 1		Model 2		Model 1		Model 2	
Risk marker		pvalue		pvalue		pvalue		pvalue
Age per decade	2.35 (1.81, 2.90)	1.2E-17	2.42 (1.87, 2.98)	4.0E-18	2.47 (2.15, 2.80)	<1.0E-300	2.44 (2.11, 2.77)	<1.0E-300
Sex (female)	4.09 (3.19, 4.99)	1.4E-19	4.26 (3.35, 5.18)	9.0E-21	1.22 (0.69, 1.74)	5.4E-06	1.68 (1.15, 2.21)	4.8E-10
Systolic BP per 10mmHg	1.21 (0.96, 1.47)	2.0E-20	1.25 (0.99, 1.51)	7.2E-21	0.61 (0.45, 0.76)	1.2E-14	0.39 (0.24, 0.55)	7.7E-07
Diastolic BP per 10mmHg	0.80 (0.36, 1.24)	3.6E-04	0.83 (0.37, 1.29)	3.8E-04	0.25 (-0.01, 0.52)	5.9E-02	-0.38 (-0.65, -0.11)	0.006
Mean arterial pressure per 10 mmHg	1.31 (0.92, 1.69)	2.5E-11	1.37 (0.97, 1.77)	1.2E-11	0.58 (0.35, 0.81)	8.3E-07	0.11 (-0.13, 0.34)	0.367
BMI per 5 kg/m²	0.20 (-0.26, 0.67)	0.400	0.21 (-0.26, 0.68)	0.384	2.46 (2.17, 2.74)	<1.0E-300	2.42 (2.13, 2.70)	<1.0E-300
Total fat mass index kg/m²	0.17 (-0.50, 0.84)	0.620	0.17 (-0.49, 0.85)	0.610	2.00 (1.59, 2.41)	3.1E-22	1.96 (1.55, 2.37)	2.3E-21
Total fat free mass index kg/m²	0.11 (-0.77, 1.00)	0.801	0.12 (-0.77, 1.01)	0.797	0.94 (0.40, 1.47)	5.6E-04	0.93 (0.40, 1.47)	6.3E-04
HbA_{1c} per 5 mmol/mol*	0.60 (0.24, 0.97)	0.001	0.55 (0.17, 0.92)	0.004	1.12 (0.90, 1.34)	8.7E-24	0.70 (0.48, 0.93)	8.2E-10
Total cholesterol (mmol/L)	0.16 (-0.23, 0.55)	0.435	0.16 (-0.23, 0.55)	0.432	-0.51 (-0.74, -0.28)	1.8E-05	-0.44 (-0.67, -0.20)	2.5E-04
HDL cholesterol (mmol/L)	0.48 (-0.77, 1.75)	0.451	0.83 (-0.52, 2.19)	0.230	-2.13 (-2.86, -1.39)	2.1E-08	0.21 (-0.59, 1.02)	0.601
LDL cholesterol (mmol/L)	-0.02 (-0.52, 0.49)	0.939	-0.03 (-0.54, 0.48)	0.900	-0.48 (-0.78, -0.17)	0.002	-0.56 (-0.86, -0.26)	3.0E-04
Triacylglycerol (mmol/L)	0.86 (0.39, 1.33)	2.9E-04	0.81 (0.33, 1.30)	0.001	0.22 (-0.05, 0.50)	0.113	-0.50 (-0.79, -0.22)	6.0E-04
CRP (µmol/L)	0.44 (0.03, 0.86)	0.036	0.39 (-0.07, 0.86)	0.097	1.42 (1.17, 1.67)	4.4E-29	0.51 (0.23, 0.79)	3.2E-04
White cell count (10^9 cells/L)	-0.30 (-0.52, -0.09)	0.005	-0.41 (-0.63, -0.20)	2.2E-04	0.33 (0.20, 0.46)	5.7E-07	0.10 (-0.04, 0.23)	0.151
Granulacytes (10^9 cells/L)	-1.33 (-2.23, -0.41)	0.005	-1.76 (-2.69, -0.82)	2.6E-04	1.80 (1.23, 2.36)	2.8E-10	0.91 (0.34, 1.49)	0.002

Percentage difference in retinal vessel tortuosity are from a multilevel model allowing for repeated images from the same person (random effect for person). Confidence interval (CI). Model 1 adjusting each factor for age, sex, ethnicity and UKBB centre as fixed effects (n= 50,233). Model 2 adjusting each factor for model 1 plus body mass index, smoking and the Townsend deprivation index, as fixed effects (n=43,888). *HbA_{1c} model 1 n=46,122 and model 2 n=46,122.
